# Food allergy and anaphylaxis - 2041. Rush oral immunotherapy for severe food allergy: one year follow up

**DOI:** 10.1186/1939-4551-6-S1-P126

**Published:** 2013-04-23

**Authors:** Mayumi Sugimoto, Mizuho Nagao, Mari Kondo, Keigo Kainuma, Takao Fujisawa

**Affiliations:** 1 Institute for Clinical Research, Mie National Hospital, Japan

## Background

Prevalence of severe food allergy with high risk of anaphylaxis has been increasing and daily restricted diet and fear of accidental anaphylaxis are great burden on the patients. Oral immunotherapy (OIT) may be a hope for the cure but has not been established at present. We performed OIT for severe food allergy with a unique protocol aiming to achieve the dosing of high amount during rush phase.

## Methods

One hundred and one children with egg, milk and/or wheat allergy were enrolled in the study. OIT protocol was consisted of initial rush phase following maintenance phase. Goals of dosing during rush phase were one half-boiled egg, 200ml of milk, one serving of wheat as a staple food for egg, milk and wheat allergy, respectively. Seventy-nine patients (since some patients received more than one OIT, number of OITs were 59 for egg, 43 for milk and 12 for wheat) who reached 12 months of maintenance were analyzed.

## Results

Percentages of cases who achieved the goal dosing were 84.5%, 78.4% and 87.5% in egg, milk and wheat OITs, respectively, during rush phase. At one year of maintenance, 92.7% of patients on egg OIT ingested one boiled-egg or half-boiled egg, 77.8% on milk OIT ingested 200ml of milk and 85.7% on wheat OIT ingested one serving of wheat as a staple food. However, 1 patient with milk OIT and 1 with wheat OIT returned to complete elimination because of suspected eosinophilic esophagitis and frequent anaphylaxis, respectively. Allergen-specific IgE and basophil activation were significantly decreased and allergen-specific IgG<sub>4</sub> was significantly elevated after rush OIT in most patients including failure cases.

## Conclusions

Our rush OIT brought desensitized state in most patients with severe food allergy. Further investigation is necessary to clarify the factors to predict the prognosis of OIT.

